# Effect of the number of negative lymph nodes removed on the survival and recurrence rate after gastrectomy in patients with gastric cancer: a multicenter retrospective cohort study

**DOI:** 10.1186/s12893-023-02154-9

**Published:** 2023-08-21

**Authors:** Mansour Bahardoust, Mahdieh Kheirabadi, Ghazaleh Donyadideh, Mohsen Khaleghian, Meisam Haghmoradi, Adnan Tizmaghz

**Affiliations:** 1 Department of Epidemiology, School of Public Health, Shahid Beheshti University of Medical Sciences, Tehran, Iran; 2 Medical student, School of Medicine, Iran University of Medical Sciences, Tehran, Iran; 3 School of Medicine, Mashad University of Medical Sciences, Mashad, Iran; 4 Department of General Surgery, School of Medicine, University of Medical Sciences, Urmia, Iran; 5 Department of Orthopedic Surgery, Urmia University of Medical Sciences, Urmia, Iran; 6 Department of General Surgery, School of Medicine, Firoozabadi Hospital, Iran University of Medical Sciences, Tehran, Iran

**Keywords:** Gastric cancer, Five- survival rate, Recurrence rate, Gastrectomy

## Abstract

**Background:**

Various factors affect the survival rate of Gastric cancer (GC) patients after gastrectomy. This study aimed to investigate the effect of the number of negative lymph nodes (NLNs) removed on GC patients’ survival and recurrence rate after gastrectomy.

**Methods:**

In this retrospective, multicenter cohort study, we reviewed the medical profile of 639 patients with a definite diagnosis of GC who underwent gastrectomy from 2010 to 2022 in one of the medical centers affiliated with the Iran University of Medical Sciences. Based on the number of NLNs removed, patients were divided into three groups, including (0–9NLNs), (10–15 NLNs), and (≥ 16 NLNs), including 155, 231, and 253 GC patients, respectively. Demographic characteristics, tumor characteristics, and pathological findings of the patients were extracted by referring to the patient’s files.

**Results:**

The 5-year survival rate of patients was estimated at 48.2%. The 5-year tumor recurrence rate in patients with the number of NLNs 1–9, NLNs 10–15, and ≥ 16 NLNs were 79.4%, 51.1%, and 30.8%, respectively. (Log-rank = 9.24, P 0.001) The multivariate analysis showed that the 5-year survival rate in patients with fewer NLNs removed ≥ 16 was significantly higher than in the other two groups. In addition, age, BMI, tumor size, tumor stage, metastasis, and tumor differentiation were significantly related to the survival of GC patients after gastrectomy. (p < 0.05)

**Conclusion:**

Paying attention to the number of NLNs removed during gastrectomy can be a key factor in improving the survival prediction of GC patients.

## Introduction

Gastric cancer (GC) is the most common malignancy worldwide [1]. 2018 according to reports from the International Agency for Research on Cancer (GLOBOCAN), 1,033,701 new GC cases (5.7% of all diagnosed cancer cases) and 782,685 GC-related deaths were recorded worldwide [2]. GC is the fifth most common cancer and the third most common cause of cancer death (8.2% of all cancer deaths) after lung and colorectal cancers [2, 3]. The incidence and mortality rate of advanced gastric cancer is different among countries, as the number of death and complications in East Asian countries are fewer than the Western countries [4]. The median survival rate is less than 12 months in the advanced stage [5, 6].

Various factors, such as tumor characteristics and demographic characteristics of patients, are related to the survival rate of GC patients after gastrectomy [7]. Studies have shown that with the increase in the number of positive lymph nodes and the presence of metastasis, the survival rate of patients decreases significantly [7, 8]. Recently, several studies have shown that, in addition to positive lymph nodes, the number of negative lymph nodes (NLNs) resected is also related to the survival rate of patients with GC [6, 9, 10]. Negative lymph node counts have attracted more attention as a prognostic factor in gastric cancer and other cancers such as colon, esophageal, and cervical cancers [11–14].The lymph node status is the most important predictor for patients with GC after surgical resection [15]. It is worth noticing that surgical resection is the crucial and only therapy chance for GC patients [15]. The NLNs have played an important role in the survival rate of GC patients after GC surgery in recent years [16]. Some studies showed that the higher the negative lymph node ratio, the better survival [6, 16]. Very limited studies have investigated the effect of the number of negative nodes on the survival rate of patients with GC. The survival rate of patients with GC is different based on the demographic characteristics and race of the patients [17, 18]. According to our knowledge, no study in Iran has investigated the effect of negative lymph node count in GC patients after surgery. This study aimed to evaluate the impact of the number of resected negative lymph nodes on the survival and recurrence rates of patients with GC after surgery.

## Methods

### Patient characteristics

**Fig. 1:**
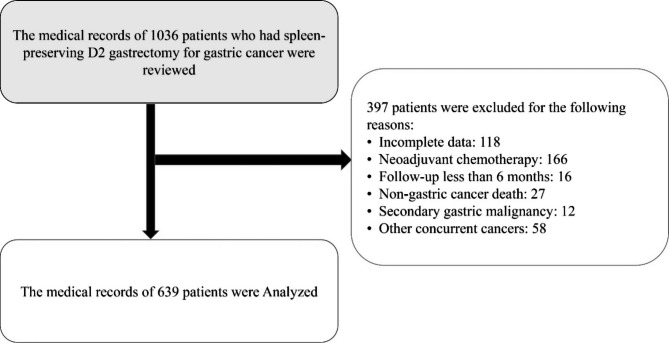
Flowchart of study

The Iran University of Medical Sciences ethics committee approved this study with code IR.IUMS.REC.1402.316. In this multi-center retrospective cohort study, the medical profile of 1036 patients with a definite diagnosis of GC who had undergone Spleen-preserving D2 lymph dissection from the beginning of 2010 to the beginning of 2022 in one of the three academic referral hospitals (tumor surgery centers) affiliated to Iran University of Medical Sciences was reviewed. We selected centers that met the study’s inclusion criteria to examine the medical profile of GC patients who underwent surgery. All three medical centers affiliated with Iran University of Medical Sciences, which specializes in cancer surgery, were chosen for the study. After applying the inclusion criteria, the medical profile of 639 consecutive patients was examined to investigate the effect of the NLNs removed and other factors on the survival rate and tumor recurrence after surgery. (Fig. 1)

The inclusion criteria for this study included: a definitive diagnosis of GC based on endoscopic and pathological findings, Patients with D2 dissection, patients treated with surgery (total or subtotal gastrectomy), patients with at least six months of follow-up, postoperative adjuvant chemotherapy and the availability of medical profiles. Receiving chemotherapy or radiotherapy before and gastrectomy, secondary GC (tumors metastasized to the stomach from other organs), patients whose main cause of death were other than GC and simultaneously suffering from other tumors or receiving treatment for other tumors were defined as exclusion criteria. The oncologist re-checked the patients’ medical profiles and made the definitive diagnosis of GC based on the pathological findings. Classification of the GC was done based on the tumor-node-metastasis (TNM) guide (tumor size (< 3 cm / >3 cm), number of nodules, (N) the number of involved lymph nodes (2 < vs.>2), and the presence of metastasis (positive/ (negative) for other organs) [7, 19, 20]. Patients were divided into three groups to estimate the effect of the NLNs removed, including patients with 1–9, 10–15, and ≤ 16 NLNs.

### Variables and data collection

The data was collected using a checklist by the researcher from the patient’s file. Demographic information of the patients included (age, gender, body mass index (BMI), education and history of smoking, and family history of GC). Tumor characteristics and pathological findings of patients include (tumor location, tumor stage, tumor size, presence of metastasis to other organs, the number of negative lymph nodes removed) and the survival rate (median survival time, number of deaths, mean follow-up, and tumor recurrence). Predictive factors of survival rate were estimated. Five-Survival, recurrence rate, and duration of recurrence surgery in three subgroups were compared with the NLNs removed. Survival was defined as the time interval between surgery and death.

### Spleen-preserving D2 lymph dissection technique

In this study, D2 gastrectomy with spleen preservation was performed for all patients with a detailed dissection of each LN station and careful handling of the spleen and its vessels to prevent injury or bleeding. This surgery is performed in the following steps based on the review of the files and interviews with the patients’ surgeons in recent years. 1- After mobilizing the stomach and dividing the gastrocolic ligament, and the gastrocolic omentum along with the stomach, the stomach was retracted to the right side and the splenic artery was identified at its origin from the celiac trunk and traced distally along its course.2- The LNs along the splenic artery (No. 11p) are dissected from its origin to its bifurcation into the dorsal pancreatic artery, and greater curvature artery.3-The splenic vein is identified at its confluence with the superior mesenteric vein and traced proximally along its course.4-The LNs along the splenic vein (No. 11d) are dissected from its confluence to its entrance into the splenic hilum.5-The splenocolic ligament is divided to free the lower pole of the spleen.6- The splenorenal ligament is divided to free the upper pole of the spleen.7- The tail of the pancreas is separated from the posterior wall of the stomach by blunt dissection.8-The SHLN (No. 10) is dissected from the splenic hilum by dividing the connective tissue between the spleen and the pancreas.9- The spleen and the pancreatic tail are returned to their original position, and hemostasis is checked [21, 22].

The details about removed lymph node stations, marking of lymph nodes, and inking of edges for D2 gastrectomy are as follows: Removed lymph node stations: D2 gastrectomy involves removing the stomach and the lymph nodes around it, including the perigastric LN (No. 1–6), the left gastric artery nodes (No. 7), the common hepatic artery nodes (No. 8a), the celiac artery nodes (No. 9), the splenic artery and vein nodes (No. 11p and 11d), and the splenic hilar nodes (No. 10). Marking of lymph nodes: The lymph nodes are marked according to their station number using different colored clips or sutures to facilitate identification and counting. The marking is done before or after removing the lymph node-bearing tissue, depending on the surgeon’s preference. Inking of edges: The edges of the resected specimen are inked with different colors to indicate the proximal, distal, greater curvature, and lesser curvature margins. The inking is done after the removal of the specimen and before sending it to pathology.

### Sample size calculation

The appropriate sample size for conducting this study, with an effect size estimate of 0.31, for the difference in the survival rate of patients with GC based on the NLNs based on the study of D Xu et al.,[23] with an alpha error of 0.05 and a power of 80%, was estimated by an epidemiologist to be 320 patients using G Power version 3.1 software.

### Statistical analysis

Data analysis was done using SPSS version 22 and Stata 17 software. Log-rank test was used to compare the survival rate of basic variables, clinical parameters, and tumor characteristics. Mean ± SD and number (%) were used to report continuous and categorical variables, respectively. Kaplan-mired, the product limit estimator, was used to estimate the survival function. Variables with a p-value of less than 0.15 in the univariate analysis were included in the Cox multivariate analysis. Multiple Cox regression with a stepwise backward selection variable strategy was used to assess the adjusted associations of the covariates and time to event. Multivariate analysis was used to control the effect of stage and other tumor characteristics on patient survival. The adjusted hazard ratio (HR) in the 95% confidence interval (95% CI) was used to determine the variables predicting survival. A P-value less than 0.5 was considered a statistically significant level.

## Results

### Demographic and tumor characteristics

**Fig. 2:**
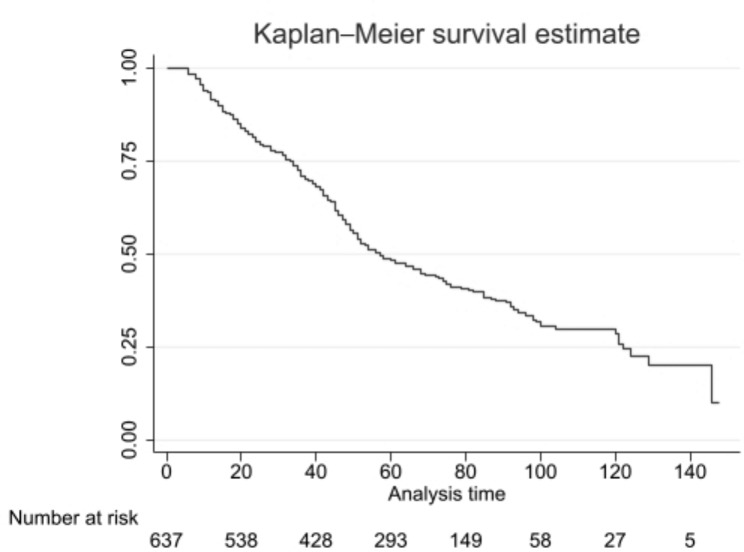
Kaplan-Meier overall 5-year survival of patients

The medical profiles of 639 patients with GC were reviewed. The mean age of the patients was 60.12 ± 11.54 years (range 32 to 86 years). The median age of the patients was 61 years. The overall mean BMI was 23.5 ± 3.25 kg/m2. Most of the patients were married. More than half of the patients were illiterate or had less than a diploma. Almost 40% of tumor cases were in the cardia region. With 343(53.7%), adenocarcinoma was the most common tumor type. The mean follow-up was 74.3 ± 23.2 months. Median survival was 59.1 months after surgery. 269 (42.1%) had tumor size < 5 cm. Metastasis was reported in 209 (32.7%) cases. Regarding the NLNs, 1 to 9 NLNs were removed in 155(24.3%) patients. 10 to 15 nodes were removed in 231(36.1%) patients, and more than 16 nodes were removed in 253(39.6%) patients. (Table 1) Overall 5-year survival of patients was estimated to be 48.2%. (Fig. 2)

**Table 1:** Demographic and pathological characteristics of GC patients

Demographic Characteristics	639 patients with NLNs GC
**Age (year) (mean ± SD)**	60.12 ± 11.54
BMI (Kg/m^2^)	23.5 ± 3.25
Mean follow-up (Month)	74.3 ± 23.2
Median survival (Month)	59.1 ± 26.2
**Sex**	
• Male • Female	338(52.9%) 301(47.1%)
**Marital status**	
• Unmarried • Married	37(5.8%) 602(94.2%)
**Smoking status**	
• Yes • NO	275(43.1%) 193(56.9%)
**Education**	
• Illiterate • < Diploma • >=Diploma	99(15.6%) 264(41.3%) 264(43.1%)
**Alcohol consumption**	
• Yes • NO • Missing	131(20.5%) 311(48.7%) 197(30.8%)
**H- pylori test**	
• positive • negative • Missing	234(36.6%) 159(24.9%) 246(38.5%)
**Family history of GC**	
• **Yes** • **No**	260(40.7%) 379(59.3%)
**Tumor Location** • Cardia • Fundus • Antrum • Missing	260(40.7%) 187(29.2%) 109(17.1%) 83(13%)
Tumor Type	
• Adenoma carcinoma • Lymphoma • Other & Unknown	343(53.7%) 60(9.4%) 236(36.9%)
Number of examined LNs	
• < 15 • ≥ 15	322(50.4%) 253(49.6%)
**Tumor size (cm)**	
• **≤ 5** • **> 5** • Unknown	269(42.1%) 310(48.5%) 64(9.4%)
**NLNs**	
• 1–9 • 10–15 • ≥ 16	155(24.3%) 231(36.2%) 253(39.6%)
**Metastasis**	
• Yes • No	209(32.7%) 430(67.3%)
**Tumor stage**	
• T1 • T2 • T3 • Unknown	128(20%) 207(32.2%) 152(23.9%) 152(23.9%)
**Differentiation grade**	
• Well • Moderate • Poor • Unknown	249(38.9%) 190(29.8%) 77(12.1%) 123(19.2%)

Survival Rate

The overall 5-year survival of patients was estimated to be 48.2%. (Fig. 2)

### Univariate analysis finding

**Fig. 3:**
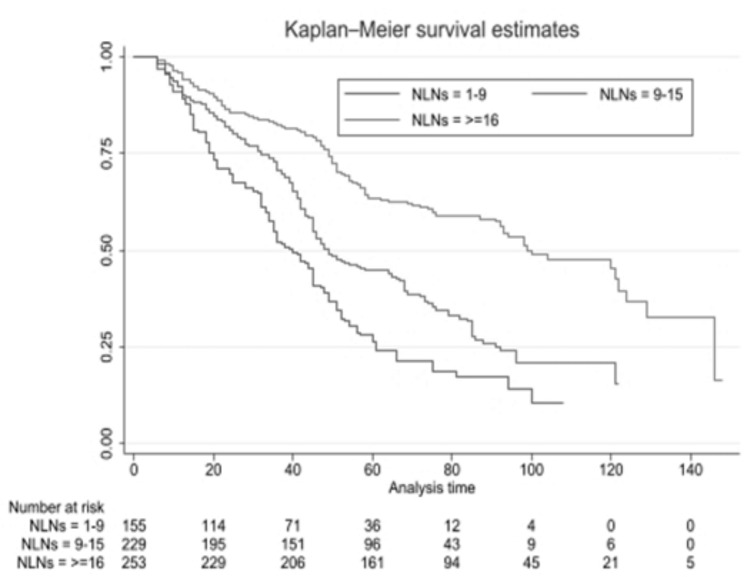
5-year survival chart of patients based on the number of NLNs removed

The 5-year survival rate in patients ≤ 60 years old was significantly better than in patients 60. The survival rate of patients with a BMI of ≤ 18 and > 25 kg/m^2^ was significantly lower than that of a BMI of 18 to 25 kg/m2. (Log-rank = 8.21, P 0.006) The number of NLNs removed is significantly related to the survival rate of patients. So, the 5-year survival rate in patients with the number of NLNs removed from 1 to 9 and 10 to 15 was significantly lower than that of patients with ≥ 16 number of NLNs removed. (Log-rank = 11.36, P 0.001) (Fig. 3) The 5-year tumor recurrence rate in patients with the number of NLNs 1–9, 10–15, and ≥ 16 was 79.4%, 51.1%, and 30.8%, respectively, and this difference was statistically significant. (Log-rank = 9.24, P 0.001)

The univariate analysis showed that tumor size above 5 cm, the presence of metastasis, tumor stage, differentiation grade, and tumor type were related to the survival of patients. (p < 0.05) (Table 2).

**Table 2:** Univariate analysis of factors predicting patient survival

Variable	Five –Survival rate	HR (95% CI)	P value
Age (year)			0.043
• ≤ 60 • > 60	59.4 37.6	Ref 1.57(1.03,2.11)	
BMI (Kg/m^2^)			
• ≤ 18 • 18.1–25 • ≥ 25	31.5% 57.9% 35.6%	1.81(1.12,2.52 Ref 1.61(1.03,2.2)	P:0.001 - 0.008
Sex			0.58
• Female • Male	46.2% 51.6%	Ref 1.09(0.75,2.11)	
Marital status			0.87
• Unmarried • Married	47.9% 48.8%	Ref 1.02(0.88,3.56)	
**Smoking status**			0.087
• **No** • **yes**	45.4% 51.6%	Ref 1.12(0.99,4.11)	
**Education**			
• Illiterate • < Diploma • >=Diploma	38.6% 45.6% 50.7%	Ref 0.85(0.64,1.07) 0.77(0.53,1.3)	- 0.061 0.083
**Alcohol consumption**			
• Yes • NO • Missing	34.4% 44.2% 45.5%	Ref 0.78(0.51,1.01) 0.75 (0.50,1.01)	- 0.089 0.084
** H- pylori history**			
• positive • negative • Missing	40.5% 47.6% 44.4%	Ref 0.85(0.64,1.08) 0.90(0.74,1.07)	- 0.075 0.11
**Family history of GC**			
• **Yes** • **No**	45.7% 49.2%	1.07(0.75,2.71) Ref	0.45
**Tumor Location**			
• Cardia • Fundus • Antrum • Missing	45.5% 47.3% 50.5% -	Ref 0.96(0.81,1.12) 0.9(0.76,1.11) -	- 0.057 0.068
Tumor Type			
• Adenoma carcinoma • Lymphoma • Other & Unknown	56.2 30.28 42.5	0.81(0.74,0.87) 0.88(0.65,1.56) Ref	0.0025 0.54 -
Number of examined LNs			
• < 15 • ≥ 15	36% 49%	Ref 0.72(0.61,0.84)	- 0.001
**NLNs**			
• 1–9 • 10–15 • ≥ 16	23.9% 47.2% 65.2%	Ref 0.53(0.36,0.71) 0.38(0.22,0.55)	- 0.004 0.001
**Tumor size (cm)**			
• **≤ 5** • **> 5** • Unknown	52.3% 45.2% 46.4%	Ref 1.21(1.01,2.65) -	- 0.064
**Metastasis**			
• Yes • No	33.5 58.2	1.75(1.16,2.36 Ref	0.001
**Tumor stage**			
• T1 • T2 • T3 • Unknown & missing	76.2% 50.2% 24.2% -	Ref 1.6(1.09,2.12) 3.2(1.56,4.83) -	- 0.001 0.001 -
**Differentiation grade**			
• Well • Moderate • Poor • Unknown & missing	64.2 50.2 23.5 -	Ref 1.28(1.08,1.49) 2.7(1.44,3.97) -	- 0.023 0.001 -

### Comparison of demographic and tumor characteristics based on the number of NLNs removed

Demographic and tumor characteristics were compared among the groups with the number of NLNs removed; the 5-year survival rate in patients with ≥ 16 NLNs removed was significantly higher than in the other two groups. (p:0.001). The median time for tumor recurrence was significantly higher in patients with ≥ 16 NLNs removed than in other groups. (P: 0.001) The presence of metastasis and poor tumor differentiation was significantly lower in the group with the number of NLNs ≥ 16 compared to the other two groups. (p < 0.05) No significant correlation was observed between the number of NLNs harvested with demographic and other tumor characteristics (p > 0.05). (Table 3)

**Table 3:** Comparison of demographic and tumor characteristics based on the number of NLNs removed

Variable	Number of NLNs resected			P value
	1–9 (n:155)	10–15 (n:231)	≥ 16 (n:253)	
**Age (year) (mean ± SD)**	61.26 ± 10.56	59.1 ± 11.61	60.18 ± 11.23	0.75
BMI (Kg/m^2^)	20.1 ± 3.12	22.8 ± 3.21	25.11 ± 3.27	0.021
Median survival (Month)	36.21 ± 11.2	49.1 ± 9.16	87.3 ± 11.3	0.001
Five- survival rat	37(23.9%)	109(47.2)%	165(65.2%)	0.001
Five- Recurrence rate	123(79.4%)	118(51.1%)	78(30.8%)	0.001
Median recurrence (Month)	19.5 ± 8.6	31.8 ± 10.5	51.5 ± 10.9	0.001
**Sex**				0.44
• Male • Female	78(50.3%) 77(49.7%)	129(55.8%) 102(44.2%)	131(52.9%) 122(47.1%)	
**Marital status**				0.54
• Unmarried • Married	10(6.5%) 145(93.7%)	12(5.2%) 219(94.8%)	15(5.9%) 238(94.1%)	
**Smoking status**				0.16
• **Yes** • **NO**	70(49%) 85(51%)	99(42.9%) 132(57.1%)	146(43.1%) 193(56.9%)	
**Education**				0.26
• Illiterate • < Diploma • >=Diploma	25(16.1%) 64(41.3%) 66(57.4%)	35(15.2%) 99(42.9%) 97(41.9%)	39(20.2%) 101(39.9%) 101(39.9%)	
**Family history of GC**				0.11
• **Yes** • **No**	62(40%) 93(60%)	98(42.4%) 133(57.6%)	100(39.5%) 153(60.5%)	
**Tumor Location**				0.058
• Cardia • Fundus • Antrum • Missing	49(31.6%) 47(30.3%) 25(16.2%) 343(21.9%)	94(40.7%) 68(29.4%) 31(13.4%) 38(16.5%)	117(46.4%) 72(28.4%) 53(20.9%) 11(4.3%)	
Tumor Type				0.061
• Adenoma carcinoma • Lymphoma • Other & Unknown	83(53.5%) 20(12.9%) 52 (33.6%)	116(50.2%) 25(10.8%) 90(39%)	109(56.9%) 15(5.9%) 94(37.2%)	
**Metastasis**				0.012
• Yes • No	62(40%) 93(60%)	78(33.7%) 153(66.3%)	69(27.3%) 184(72.7%)	
**Tumor size (cm)**				0.16
• **≤ 5** • **> 5** • Unknown	70(45.1%) 74(47.7%) 11(7.2%)	94(40.7%) 111(48.1%) 26(11.2%)	101(39.9%) 125(49.4%) 27(10.7%)	
**Tumor stage**				0.054
• T1 • T2 • T3 • Unknown	32(20.6%) 48(31%) 40(25.8%) 35(22.6%)	43(18.6%) 69(29.8%) 81(35.1%) 38 (16.5%)	53(20.9%) 90(35.6%) 31(12.3%) 79(31.2%)	
**Differentiation grade**				0.001
• Well • Moderate • Poor • Unknown	43(27.7%) 50(32.3%) 20(12.9%) 42(27.1%)	85(36.8%) 70(30.3%) 35(15.2%) 41(17.7%)	121(47.8%) 70(27.6%) 22(8.9%) 40(15.7%)	

### Cox multivariate analysis finding

To determine the effect of the number of NLNs removed on survival adjusted for other tumor and demographic variables, all variables with a p-value less than 0.15 in the univariate analysis were entered into the Cox multivariate analysis adjusted with the Backward methods. The results of multivariate analysis showed that the number of NLNs removed was significantly related to the 5-year survival of patients, and the 5-year survival was significantly higher in patients with the number of NLNs removed ≥ 16 compared to the other two groups. Multivariate analysis showed that patients’ age, mean BMI, tumor size, tumor stage, presence of metastasis, and pathological tumor differentiation were significantly related to the survival of patients with GC (P < 0.05). (Table 4)

**Table 4:** Independent predictors for cancer-specific survival by Cox multivariate analysis finding

Variable	HR _adjusted_	95% CI	P value
Age (> 60 Vs < 60 year)	1.41	1.02,1.81	0.001
BMI (Kg/m^2^)			
• ≤ 18 • 18.1–25 • ≥ 25	1.59 Ref 1.43	1.09,2.2 1 1.02,186	0.004 - 0.008
**Tumor size (> 5 vs. ≤ 5 cm (**	1.19	1.01,1.42	0.034
**Metastasis (Presence and absence)**	1.71	1.12,2.3	0.001
**NLNs** • 1–9 • 10–15 • ≥ 16	Ref 0.68 0.51	Ref 0.46,0,91 0.39,0.63	- 0.001 0.001
**Tumor stage**			
• T1 • T2 • T3	Ref 1.53 2.87	- 1.04, 2.1 1.19,4.56	- 0.026 0.001
**Differentiation grade**			
• Well • Moderate • Poor	Ref 1.23 2.61	- 1.05, 1.41 1.21, 4.01	- 0.003 0.001

## Discussion

The survival of patients with GC after gastrectomy depends on various factors, including the patient’s age and the tumor’s characteristics, such as metastasis, tumor size, and the number of lymph nodes. Although many studies have examined the factors predicting the survival of patients with GC[7, 24, 25], the number of studies that have examined the effect of the number of NLNs removed in gastrectomy on the survival and recurrence rate of GC patients is limited. According to our knowledge, no study has investigated the effect of the number of NLNs removed in gastrectomy on the survival and recurrence rate of Iranian patients with gastric cancer. Although many studies have investigated predictors of the survival of patients with GC, studies that have investigated the effect of the number of negative lymph nodes removed in gastrectomy on the survival rate of patients are limited. Therefore, considering the importance of the problem and the high prevalence of GC, we evaluated the effect of the number NLNs harvested on the survival rate and recurrence of patients with GC who had undergone D2 gastrectomy techniques.

Based on our study’s results, most tumors were of the squamous type, the patients were in the first and second stages of the disease, and metastasis had occurred in nearly 35% of the patients. The median survival was almost 60 months, which shows that the survival rate in surgery is higher than in chemotherapy and radiotherapy. The 5-year survival and recurrence rate after gastrectomy was significantly related to the number of NLNs harvested during gastrectomy. In patients with ≥ 16 NLNs removed during gastrectomy, the survival rate was significantly higher, and the recurrence rate was significantly lower. Also, the 5-year survival rate in patients with the number of NLNs removed between 10 and 15 was higher than the others with < 10 removed lymph nodes. The multivariate analysis showed that the number of NLNs removed, age of patients, mean BMI, tumor size, tumor stage, the presence of metastasis, and differentiation of tumor were significantly associated with the survival of patients with GC, which that the results of our study were consistent with the results of studies conducted in this field [9, 10, 23]. R Biffi et al.,[10] by examining the effect of the number NLNs removed on the survival rate of 114 patients with GC with a median age of 63 years, showed that the number of NLNs removed was significantly associated with patient survival after Gastrectomy. The survival rate was significantly higher in the group of patients with the number of NLNs removed > 15 compared to ≤ 15 in gastrectomy. In their study, they reported that in addition to the number of NLNs, metastasis, age of patients, tumor size, and tumor histology were significantly related to the survival of patients, which was consistent with the results of our study. In another study by D Xu et al.,[23]v by examining the effect of the number of NLNs removed on the survival rate of 435 patients with GC, showed that, in addition to tumor size, invasion depth, and tumor stage, the number of NLNs removed in gastrectomy was also related to survival rate. The survival rate in patients with the number of removed NLNs ≥ 16 was significantly higher than in other groups. Also, the survival rate in patients with the number of NLNs 15 − 11 was significantly higher than in the group with the number of removed NLNs 1–6 and 7–10. As well as in the other study which was conducted by “AJ Greenstein et al.“ [11] to assess the effect of the number of removed lymph nodes on survival in esophageal cancer, their results indicated that the survival rate in patients with more than 18 and 11–17 was higher than the others with less than ten removed lymph nodes, which is in line with our results.

One of the main findings of this study was the association between the number of NLNs removed during gastrectomy and the survival/recurrence rates of GC patients. This association may have several biological or clinical explanations. First, NLNs may reflect the extent of lymphadenectomy and the quality of surgical technique, as more NLNs indicate a more thorough dissection of the lymphatic basin and a lower risk of residual disease[26, 27]. Second, NLNs may have a protective role in preventing cancer spread by acting as barriers or filters that trap tumor cells and prevent them from reaching distant sites [26, 28]. Third, NLNs may enhance the immune response against tumor cells by producing cytokines or presenting antigens that activate cytotoxic T cells and natural killer cells[26–28]. However, it is also possible that some patients with low NLN counts may have been misclassified as node-negative due to insufficient sampling or detection of micrometastases, which may affect the survival outcomes [29].

In a systematic review, R Seevaratnam et al. [30]showed that harvesting more LNs during gastrectomy can lead to fewer metastases to other organs and possibly better long-term outcomes. However, the number of negative lymph nodes is still unclear. In another study, Jingyu Deng et al.[9] examined 456 patients with GC. They demonstrated that the survival rate in patients with a number of NLNs > 15 was significantly higher than in patients with a number of NLNs removed < 15. They showed that paying attention to the number of NLN removed at the time of surgery is a key factor for improving the survival of patients with GC, which was consistent with the results of our study. Also, in another study which was conducted by " He, W.Z. et al.“ [31] and the effect of the number of negative lymph nodes on survival in colon cancer patients was assessed, it has revealed that the number of NLNs led to increasing the immune response and consequently enhanced the survival rate in this patients which confirmed our results. Our findings also revealed that variables like age, BMI, tumor size, metastasis (Presence and absence), Tumor stage, and Differentiation grade are significantly related to overall survival in patients’ GC who had undergone D2 gastrectomy techniques. The D2 gastrectomy techniques have changed over the years, such as Using minimally invasive approaches, such as laparoscopic or robotic surgery, to perform D2 gastrectomy. These approaches may offer advantages such as reduced blood loss, pain, and hospital stay, as well as better visualization and dexterity. However, they may also have drawbacks, such as longer operative time, higher cost, and limited availability. Spleen-preserving techniques, such as in situ or ex-situ dissection of the splenic hilar lymph nodes (SHLN), are used to avoid splenectomy during D2 gastrectomy [32–34]. These techniques may reduce postoperative morbidity and preserve the immunologic function of the spleen. However, they may also require more meticulous dissection and careful handling of the spleen and its vessels to avoid injury or bleeding. Bursectomy, which removes the peritoneal lining between the pancreas and the posterior wall of the stomach, is used to expose more lymph node stations during D2 gastrectomy. Bursectomy may improve the accuracy of staging and reduce peritoneal or local recurrence. However, it may also increase the risk of pancreatic or vascular injury. The “D2-plus” gastrectomy is a surgical procedure that involves removing the suprapancreatic LN (No. 13), the common hepatic artery LN (No. 8p), and the celiac artery LN (No. 9) in addition to treating GC situated in the antrum.“D2-plus” gastrectomy may improve survival outcomes for selected patients with advanced gastric cancer. However, it may also require more extensive dissection and increase operative morbidity [32–34].

In our study, a BMI < 18 kg/m2 was associated with decreased patient survival rate in addition to these factors. This can be justified due to anorexia, weakness, and poor health status of underweight patients, which can weaken the immune system. Several previous studies showed that a BMI of less than 18 was associated with decreased survival rates for patients with gastric ulcers after surgery. [7, 35]. Therefore, post-surgery patients’ BMI can be considered an essential independent prognostic factor.

The association between NLNs count and survival/recurrence rates has important clinical implications for managing and treating GC patients. The results suggest that ensuring adequate NLNs removed during gastrectomy may improve survival and reduce the recurrence rates of patients with node-negative or node-positive gastric cancer[26, 27, 36]. Moreover, the results help to identify patients who may benefit from adjuvant therapy or more intensive follow-up based on their NLN count and nodal ratio. For example, patients with low NLN counts and high nodal ratios may have a higher risk of recurrence or metastasis and may require more aggressive treatment or surveillance than patients with high NLN counts and low nodal ratios. Therefore, the NLN count and nodal ratio assessment may provide valuable prognostic information for GC patients and guide individualized therapeutic decisions [27, 28].

In recent years, changes in the TNM staging system and the introduction of new staging systems based on finding different prognostic factors can affect the accuracy and interpretation of the results. The current AJCC staging system does not determine the minimum number of total lymph nodes removed for adequate staging. Still, it recommends that approximately 15 LNs should be observed for radical gastrectomy. Increasing the cut-off point of total lymph nodes removed can help to improve the discrimination ability and predictive accuracy of the staging system, so designing prospective studies to evaluate the diagnostic accuracy of this TNM staging system based on different cut points is recommended.

Our study had limitations and strengths that should be noted. Designing the study retrospectively and extracting data from the patient’s medical records was the most important weakness of our study, which caused us to be unable to measure a number of important and effective factors and markers of cancer. In addition to the reason for reviewing the patient’s medical records, there were missing or unknown cases in a number of variables, such as tumor characteristics, patients with Neoadjuvant therapy, and competitor risk, which can affect the estimation of the results. Other study limitations were that multiple teams operated on patients, pathologists changed over time, guidelines changed over time, and information on treatment options (radical resection or palliative therapy), subsequent treatment, comorbidities, and recurrence was lacking. Prospective studies with a large sample size examining patients’ demographic, nutritional, and clinical characteristics to evaluate the effect of NLN removal on the survival rate and recurrence of patients with GC can help improve the survival rate of patients with GC. The results of these studies can provide a clearer view to tumor surgeons about the survival of patients after surgery. This retrospective study was conducted on Iranian patients, which limits its generalizability to other races and populations; conducting studies in different races and comparing them helps clarify the role of the number of negative nodes found in GC patients. Designing a comprehensive multicenter study with a high sample size, for the first time on the Iranian population, to evaluate the effect of the number of NLNs removed on the survival and recurrence rate of GC patients in conditions adjusted for other variables was the most important strength of our study.

## Conclusion

The results of our study have revealed that the number of NLNs resected ≥ 16 was associated with an increased survival rate and a decreased recurrence rate in GC patients after gastrectomy. Counting and paying attention to the number of NLNs removed during gastrectomy can be a key factor in improving the survival prediction of GC patients.

## Data Availability

The datasets generated or analyzed during the current study are available from the corresponding author upon reasonable request.

## Abbreviations

GC: Gastric cancer
NLNs: Negative lymph nodes
TNM: Tumor-node-metastasis
BMI: Body Mass Index
HR: Hazard ratio
CI: Confidence interval
